# Real-world outcomes of early-stage HER2-positive breast cancer patients treated with adjuvant paclitaxel and trastuzumab

**DOI:** 10.1038/s41523-025-00846-4

**Published:** 2025-11-26

**Authors:** Giorgio Bonomi, Gaia Griguolo, Tommaso Giarratano, Francesca Zanghì, Davide Napetti, Cristina Falci, Federica Miglietta, Massimo Ferrucci, Giovanni Faggioni, Grazia Maria Vernaci, Michele Bottosso, Aleix Prat, Maria Vittoria Dieci, Valentina Guarneri

**Affiliations:** 1 Department of Surgery, Oncology and Gastroenterology, University of Padova, Padova, Italy; 2 Division of Oncology 2, Istituto Oncologico Veneto IRCCS, Padova, Italy; 3 Breast Surgery Unit, Istituto Oncologico Veneto IRCCS, Padova, Italy; 4 Translational Genomics and Targeted Therapies in Solid Tumors, August Pi I Sunyer Biomedical Research Institute (IDIBAPS), Barcelona, Spain; 5 Medicine Department, University of Barcelona, Barcelona, Spain; 6 Cancer Institute and Blood Disorders, Hospital Clinic, Barcelona, Spain; 7 Reveal Genomics, Barcelona, Spain

**Keywords:** Cancer, Oncology

## Abstract

Weekly paclitaxel and trastuzumab (APT regimen) represents the standard treatment for most stage I HER2+ breast cancer (BC) patients based on results of the single arm phase II APT trial. Confirmation of long-term outcomes in real-world cohorts is of interest. This retrospective study included patients with early HER2 + BC (pT ≤ 3 cm; pN0/N1mic) treated with APT regimen. This study included 276 patients; most presented hormone receptor (HR) positive (75%, *N* = 207) and grade 3 tumors (65.6%, *N* = 181). The majority had pT ≤ 2 cm (92.4%, *N* = 255), no nodal involvement (93.1%, *N* = 257), with only 19 patients (6.9%) presenting N1mic. Anatomical stage was: IA 86.2% (*N* = 238), IB 6.2% (*N* = 17), and IIA 7.6% (*N* = 21). At a median follow-up of 4.4 years, 3-year recurrence free survival (RFS) was 97.3% (95% CI 95.1–99.5), 3-year distant relapse free survival (DRFS) was 98.2% (95% CI 96.4–100), and 3-year invasive breast cancer free survival (IBCFS) rate was 97.1% (95% CI 94.7–99.5). A statistically significant difference in RFS was observed according to anatomical stage ( *p* < 0.001). This real-world study confirms that APT regimen is associated with excellent outcomes in stage IA HER2 + BC patients, while caution is warranted for patients with stage IB or IIA disease.

## Introduction

Breast cancer (BC) is a leading cause of cancer-related deaths among women worldwide. Overexpression/amplification of the human epidermal growth factor receptor type 2 (HER2) occurs in approximately 15–20% of invasive BCs and has historically been associated with a worse disease prognosis due to a greater biological aggressiveness of this disease^1^.

The current standard of care for non-metastatic HER2 + BC includes chemotherapy in combination with HER2-targeting agents, based on the results of adjuvant pivotal trials showing that the addition of trastuzumab for one year to polychemotherapy leads to a significant reduction in the risk of relapse and death^2–5^. These pivotal studies mainly included patients with stage II-III HER2 + BC who were treated with polychemotherapy (e.g., sequence of anthracycline and taxanes or a combination of carboplatin-docetaxel). However, approximately 35–40% of all HER2 + BC cases are diagnosed as stage I and generally present a better prognosis^6^. In this context, numerous studies have investigated the possibility of using de-escalated chemotherapy and anti-HER2 regimens in patients with early HER2 + BC with favorable prognostic characteristics in order to reduce toxicities^7–9^.

Specifically, the APT regimen has become the standard treatment for most stage I HER2 + BC patients, based on the results of the APT trial^10,11^. The APT trial is a phase II single-arm study that enrolled 406 patients with HER2 + BC in the US-only, with tumors measuring up to 3 cm in greatest dimension and no lymph node metastases (pN0/pN1mic). These patients received weekly paclitaxel for twelve weeks in combination with trastuzumab, which was continued to complete one year of treatment. The study showed optimal results in terms of 3-year rate of recurrence-free survival (RFS) (99.2%; 95% CI 98.4–100) and of 3-year invasive disease-free survival (IDFS) (98.7%; 95% CI 97.6–99.8)^10^. Furthermore, the APT study demonstrated favorable long-term outcomes at 10 years. Specifically, 10-year IDFS was 91.3% (95% CI, 88.3–94.4), 10-year recurrence-free interval (RFI) was 96.3% (95% CI, 94.3–98.3), 10-year overall survival (OS) was 94.3% (95% CI, 91.8–96.8), and 10-year breast cancer–specific survival (BCSS) was 98.8% (95% CI, 97.6–100). These results further support the efficacy of the APT regimen^12^.

However, the APT trial presents some limitations; in particular, due to the single-arm phase II study design, lacking a control arm, the potential selection of a cohort of patients with a favorable prognosis cannot be excluded. Moreover, in the ATEMPT trial, which randomized patients with stage I HER2 + BC to receive adjuvant T-DM1 or the APT regimen, among 114 patients treated with the APT regimen, 9 experienced iDFS events consistent with a 5-year iDFS of 91.1% (95% CI, 85.7–96.8), and 5-year RFI of 93.2% (95% CI, 88.5–98.2)^13^.

Therefore, confirming the long-term outcomes of this regimen in independent cohorts with similar clinical characteristics is warranted.

## Results

### Patients characteristics

This monocentric retrospective study included 276 patients (273 female and 3 male) diagnosed with early-stage HER2 + BC (pT ≤ 3 cm; pN0/N1mic) treated with APT regimen. The median age at BC diagnosis was 56 years (range 26-83 years), with 181 patients (65.6%) being postmenopausal at the time of BC diagnosis.

Most patients presented tumor size pT ≤ 2 cm (92.4%, *N* = 255), with a median tumor size of 1.1 cm (range 0.1–3.0 cm). The large majority of patients had no nodal involvement (93.1%, *N* = 257), with only 19 patients (6.9%) presenting micrometastatic nodal disease (pN1mic). Anatomical stage distribution was: stage IA 86.2% (*N* = 238), IB 6.2% (*N* = 17), IIA 7.6% (*N* = 21).

Overall, 207 patients (75.0%) had a HR positive disease, with a median of ER expression of 90% (range 0–100%) and a median of PR expression of 10% (range 0–100%), and 181 (65.6%) presented a poorly differentiated tumor (G3). The majority of patients (*N* = 181, 65.6%) presented tumors categorized as HER2 IHC 3+ score, while 95 patients (34.4%) presented tumors HER2 IHC 2+ with ISH amplification.

Main clinicopathological characteristics are reported in Table 1.

**Table 1:** Clinicopathological characteristics of patients included in the study cohort (*N* = 276)

	N	%
Median age, years (range)	56 (26–83)	
Menopausal state		
Premenopausal	92	33.3%
Postmenopausal	181	65.6%
NA (male)	3	1.1%
Histology		
NST	247	89.5%
Lobular	9	3.3%
Other	20	7.2%
Histological grade		
G1	7	2.5%
G2	87	31.5%
G3	181	65.6%
NA	1	0.4%
HR status		
HR positive	207	75%
HR negative	69	25%
HER2 status		
HER2 3+	181	65.6%
HER2 2 + /ISH amplified	95	34.4%
pT		
PT1a	42	15.2%
pT1b	86	31.2%
pT1c	127	46.0%
pT2	21	7.6%
pN		
pN0	257	93.1%
PNmic	19	6.9%
Clinical stage		
IA	238	86.2%
IB	17	6.2%
IIA	21	7.6%

*NST* non special type, *HR* hormone receptor, *pT* pathologic tumor stage, *pN* pathological node stage.

### Treatments received

Breast-conserving surgery was the most frequently used surgical procedure for primary tumor treatment (66.6%). Consistently, adjuvant radiotherapy was performed in 64.5% of patients (*N* = 178).

The mean number of adjuvant weekly paclitaxel and trastuzumab cycles received was 11.74 (range 1–12). Trastuzumab was administered weekly during the chemotherapy period, and then subsequently it was administered every three weeks. The overall median number of three-weekly trastuzumab cycles, following completion of weekly treatment, was 12 (range 3–14). Only 4 patients (1.4%) discontinued trastuzumab due to cardiac toxicity.

In the subgroup of patients with triple-positive BC, aromatase inhibitors (co-administered with LHRH analogs when clinically indicated) were employed in 50.8% of cases, while tamoxifen was used in 14.1% of patients; the remaining patients received both tamoxifen and aromatase inhibitors as endocrine treatment, with a switch strategy.

### Clinical outcomes

At a median follow-up of 4.4 years (IQR 4–4.8 years), seven patients experienced a distant recurrence of the HER2 + BC (one of them also experienced a locoregional recurrence), and two of them died of HER2 + BC. One patient presented a locoregional recurrence of the HER2 + BC, was synchronously diagnosed with metastatic lung cancer, and ultimately died of lung cancer. Three additional patients were diagnosed with a locoregional recurrence, which was radically treated and did not present further BC-related events during the follow-up period. One patient was diagnosed with a contralateral HER2- BC during the follow-up period.

**Fig. 1:**
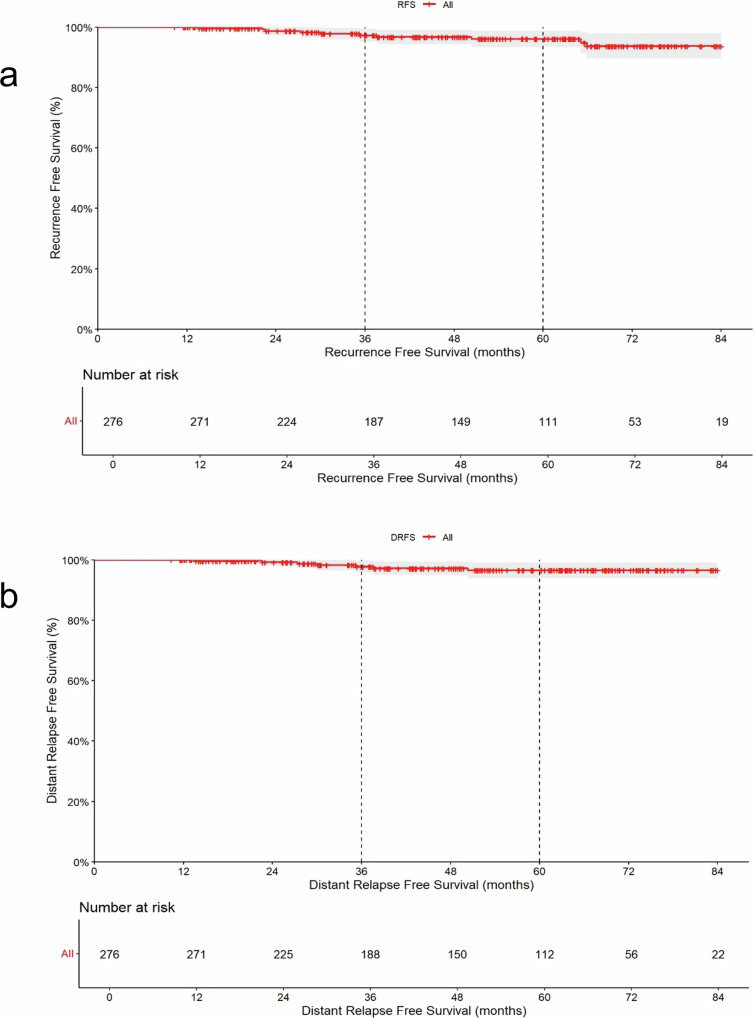
Kaplan-Meier curves showing RFS and DRFS (dashed line highlighting 36 and 60 months). **a** Kaplan-Meier curve reporting RFS. **b** Kaplan-Meier curve reporting DRFS.

The 3-year RFS rate was 97.3% (95% CI, 95.1–99.5), 5-year RFS rate was 94.9% (95% CI, 91.4–98.5; Fig. 1a), the 3-year DRFS rate was 98.2% (95% CI, 96.4–100) and the 5-year DRFS rate was 96.5% (95% CI, 94–99.1; Fig. 1b). The 3-year IBCFS rate was 97.1% (95% CI, 94.7–99.5) and the 5-year rate was 94.2 (95% CI, 90.5–97.9; Supplementary Fig. 1).

### Prognostic factors

The prognostic impact of clinicopathological features on RFS and DRFS was evaluated.

**Fig. 2:**
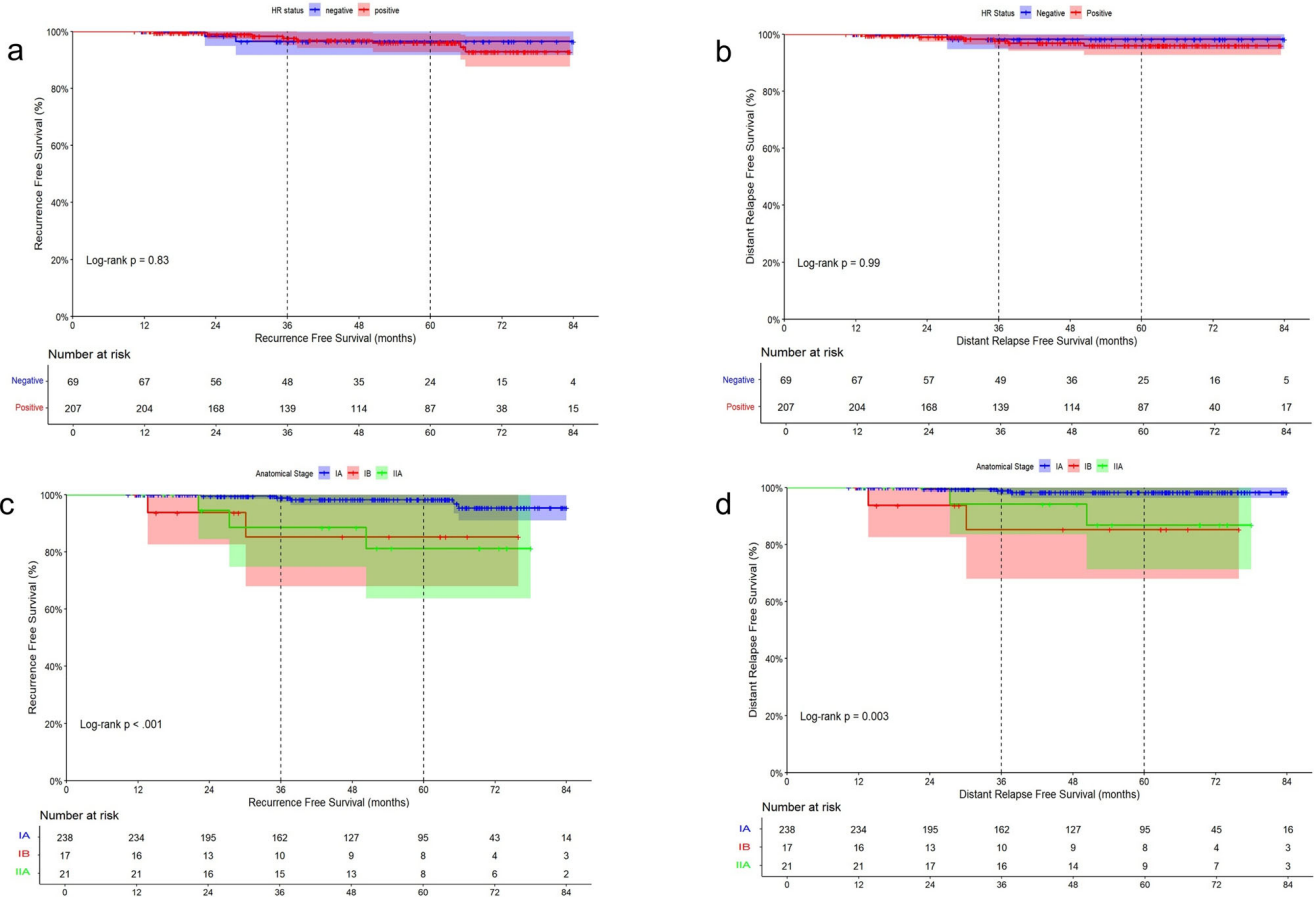
Kaplan-Meier curves showing RFS and DRFS (dashed line highlighting 36 and 60 months) according to HR status and anatomical stage. **a** Kaplan-Meier curve reporting RFS according to HR status. HR negative in blue and HR positive in red. **b** Kaplan-Meier curve reporting DRFS according to HR status. HR negative in blue and HR positive in red. **c** Kaplan-Meier curve reporting RFS according to anatomical stage. Stage IA in blue, stage IB in red, stage IIA in green. **d** Kaplan-Meier curve reporting DRFS according to anatomical stage Stage IA in blue, stage IB in red, stage IIA in green.

No significant difference in outcomes was observed according to HR status (positive vs. negative) (Table 2, Fig. 2a, b), grade, HER2 status (IHC 3+ vs 2 + /amplified), and menopausal status (Table 2, Supplementary Fig. 2).

**Table 2:** Prognostic impact of clinicopathological features on RFS and DRFS (univariate Cox models)

Clinicopathological Features		Recurrence Free Survival (RFS)			Distant Relapse Free Survival (DRFS)		
		3-year rate	HR (95% CI)	*p*-value	3-year rate	HR (95% CI)	*p*-value
**Menopausal status**	**pre-menopausal**	98.8 (96.4–100)	Ref	0.780	98.6 (95.9–100)	Ref	0.20
	**post-menopausal**	96.4 (93.3–100)	1.18 (0.3–3.9)		96.4 (91.5–99.5)	2.74 (0.58–12.97)	
**HR status**	**positive**	97.6 (95.2–100)	Ref	0.83	97.6 (95.3–99.9)	1 (0.44–2.24)	0.99
	**negative**	96.5 (91.6–100)	1.16 (0.3–4.4)		98.2 (94.6–100)	Ref	
**Grade**	**G1-G2**	100% (100–100)	Ref	0.19	100% (100–100)	Ref	0.31
	**G3**	96.1%(93–99.2)	3.9(0.5–31.22)		97.4 (94.8–99)	2.3 (0.36–24.01)	
**HER2**	**HER2 2** +	100% (100–100)	Ref	0.11	100% (100–100)	Ref	0.30
	**HER2 3** +	96.1 (92.9–99.2)	0.47 (0.20–1.31)		96.7 (93.6–99.8)	0.57 (0.20–1.63)	
**Stage**	**IA**	98 (98.3–100)	Ref	**< 0.001**	98.9 (97.3–100)	Ref	**0.003**
	**IB**	85.2 (66–100)	5.20 (0.99–27.3)		85.2 (66.2–100)	5.87(1.05–32.79)	
	**IIA**	88.5 (73.6–100)	8.15 (2.18–30.43)		94.1 (73.6–100)	8.47(1.83–39.18)	

*HR* hazard ratio, *HR status* hormone receptor status, *G* Histological grade, statistically significant p-values are reported in bold.

On the contrary, a more advanced anatomical stage was significantly associated with a worse prognosis in terms of RFS, with a 3-years RFS rate of 98% (95% CI, 98.3–100) for stage IA tumors, 85.2% (95% CI, 66–100) for IB tumors and 88.5% (95% CI, 73.6–100) for IIA tumors (log-rank *p* < 0.001) (Table 2**;** Fig. 2c). Similarly, a statistically significant difference in DRFS was observed according to anatomical stage, with 3-year DRFS rate of 98.9% (95% CI, 97.3–100) for stage IA tumors, 85.2% (95% CI, 66.2–100) for stage IB tumors and 94.1% (95% CI,73.6–100) for stage IIA (log-rank *p* = 0.003) (Table 2; Fig. 2d). Only a trend towards a worse RFS (*p* = 0.058) was observed for patients with tumor size above 1 cm when compared to patients with tumors up to 1 cm (T > 1 cm vs T ≤ 1 cm), while no significant difference in DRFS was observed between these two subgroups of patients (*p* = 0.170) (Supplementary Fig. 3). However, the trend appeared to be mainly guided by the negative impact of tumors above 2 cm, as no difference in either DFS and DRFS was observed according to tumors size when focusing on patients with tumors up to 2 cm (*p* = 0.20 and *p* = 0.41 respectively; Supplementary Fig. 4).

### Cardiac toxicity

In our study cohort, 5 patients (1.8%) experienced significant cardiac toxicity leading to trastuzumab discontinuation. In particular, only 2 patients (0.72%) presented a symptomatic grade 3 cardiac toxicity: in one case a grade 3 heart failure event (associated with pulmonary oedema) and the other case a grade 3 decrease LVEF, which presented with dyspnoea. In the remaining 3 patients, an asymptomatic but clinically relevant decline in LVEF was observed. All 5 patients discontinued treatment with trastuzumab after identification of the cardiac toxicity and details regarding the dynamic trajectories of LVEF recovery after trastuzumab discontinuation are reported in Supplementary Table 1. All patients who experienced a cardiac toxicity were over 65 years old and presented additional risk factors such as hypertension, diabetes, and/or hypercholesterolemia.

## Discussion

In this real-world cohort of patients with HER2 + BC, treated with upfront surgery followed by adjuvant paclitaxel and trastuzumab, we observed a 3-year RFS rate of 97.3% and 3-year DRFS rate of 98.2%. Overall, the treatment was well tolerated, with a trastuzumab discontinuation rate due to cardiac toxicities of less than 2%.

Overall, these results confirm that the use of the APT regimen in patients with Stage I HER2 + BC is associated with a good prognosis. Indeed, while several studies in the adjuvant setting have shown that adding trastuzumab to chemotherapy significantly improves outcomes compared to chemotherapy alone^3,14^ most of the patients enrolled in these studies presented T > 2 cm tumors and/or lymph node involvement, and an anthracycline-taxane based chemotherapy was generally used.

These pivotal studies did not include patients with smaller (T1a-T1b) tumors with negative lymph nodes, as these were considered to have a good prognosis. However, HER2 represents a powerful negative prognostic factor even for patients with T1a-b tumors^15^, and even patients with small HER2+ BCs with negative lymph nodes might potentially benefit from the addition of trastuzumab to chemotherapy, highlighting the need for specific clinical trials evaluating tailored adjuvant regimens for this patient population^16^. In this context, the APT trial, a phase II single-arm study, specifically assessed a shorter single-agent chemotherapy regimen (paclitaxel 80 mg/m² weekly for 12 weeks) in association with trastuzumab for one year, in 406 patients with HER2+ tumors ≤3 cm and no lymph node macrometastases. This study included a relatively low-risk patient population, with 41.6% of patients presenting tumors ≤1 cm and 98.5% of patients with no nodal involvement, and reported a 3-year rate of IDFS of 98.7% (95% CI, 97.6–99.8%)^10^. Even with a relatively short median follow-up of 4 years, these results led to the rapid endorsement of the APT regimen by international guidelines^11^, and the good long-term outcomes of these patients were then further confirmed at a 10-year update. Notably, these long-term outcomes were observed in APT regardless of tumour size (≤1 cm versus >1cm), HR expression, or tumor infiltrating lymphocytes levels^12^.

However, the single-arm design of the APT study raised potential concerns regarding its external validity and whether its results would be reproducible in separate cohorts. This concern was further reinforced by the observation that the small cohort of 114 patients treated with the APT regimen in the phase II ATEMPT trial showed numerically worse outcomes, with a 3-year IDFS of 93.4% (95% CI 88.7–98.2) and a 3-year RFI of 94.3% (95% CI 89.9–98.8)^17^.

A limited number of real-world studies have evaluated the effectiveness of the APT regimen over the years. Indeed, a retrospective analysis of 173 patients with HER2 + BC treated with adjuvant paclitaxel plus trastuzumab first reported a 3-year disease-free survival (DFS) and recurrence-free interval (RFI) rate of 96.6%^18^. However, this study included a significant number (12.7%) of patients with tumors larger than 3 cm and therefore, was not completely superimposable with the patient population enrolled in the APT trial and for which the APT regimen is used in clinical practice, thus making its results more difficult to interpret. More recently, a larger real-world study including 240 patients with stage I HER2 + BC reported a 3-year rwDFS rate of 98.8% with the use of the APT regimen. However, this study strictly included patients with a tumor size between 5 and 20 mm and only included 7 patients with pN1mi tumors. Therefore, the potential prognostic impact of larger tumors (2.1–3 cm) or pN1mi involvement could not be evaluated in this study^19^.

In this context, the present study represents, to our knowledge, the largest study assessing the effectiveness of the APT regimen in a real-world patient cohort, and our results generally confirm that the use of the APT regimen in patients with Stage I HER2 + BC is associated with a good prognosis.

Moreover, the 3-year RFS rate observed in our study (97.3%) was higher than the 3-year IDFS reported for patients treated with the APT regimen in the ATEMPT trial (93.4%). This difference may be partly attributable to the relatively small sample size (*N* = 114) of the APT subgroup in the ATEMPT trial, which could have introduced stochastic variability.

Patients included in our study exhibit clinicopathological characteristics generally comparable to those observed in the APT trial; however, it is important to note that there were more HR-positive patients (75% vs. 67%), a higher proportion of patients with lymph node micro-involvement (6.9% vs. 1.5%), and a greater number of patients with poorly differentiated carcinoma (65.5% vs. 56.2%).

These differences likely reflect the higher-risk and more heterogeneous characteristics of patients treated in real-world clinical practice compared with the highly selected population included in the APT trial, and may therefore potentially lead to the observation of relatively higher event rates. Therefore, outcomes observed with the APT regimen in our study cohort should be interpreted taking into account these differences in patient populations.

Moreover, it should also be highlighted that, while in the APT study, patients who had one lymph-node micrometastasis could be included if an axillary dissection was completed and no further lymph-node involvement was detected, in our patient cohort axillary dissection was not routinely performed in patients with lymph-node micrometastasis, due to changes in surgical management guidelines over time. Similarly to what was previously reported in the APT trial, we did not observe significant differences in long-term outcomes based on HR status (positive vs. negative), grade, HER2 status (IHC 3+ vs 2 + /amplified), or menopausal state. However, we did observe a significantly worse prognosis in the small subgroup of patients with more advanced anatomical stage, with a 3-year RFS rate of 85.2% (95% CI 66-100) for patients with IB tumors and a 3-year RFS rate of 88.5% (95% CI 73.6- 100) for patients with IIA tumors. These results need to be interpreted with caution, taking into account the limitations of the present study, such as the small number of patients with these characteristics included in the study, the relatively short follow-up period, and the retrospective observational design of the study. Indeed, a potential selection bias cannot be ruled out, as patients with more advanced anatomical stages who received a de-escalated treatment with the APT regimen might potentially be enriched for patients presenting prognostically negative features, such as older age, more comorbidities, and worse performance status. Nevertheless, only a minimal impact of these factors on the long-term outcomes evaluated in the study can be expected, as the majority of events observed in our study were related to the HER2 + BC of interest, and only one contralateral HER2- BC and one breast cancer-unrelated death were reported.

Despite these limitations, our results highlight the need for caution in extending the use of the APT regimen to patients with pN1mi HER2 + BC or tumors ≤ 3 cm. While the most recent guidelines do not recommend the use of the APT regimen for patients with larger tumors (2.1–3 cm), as a 20 mm cut-off is generally used for neoadjuvant treatment choice, micrometastatic lymph node involvement would generally be detected at pathological evaluation after upfront surgery, and its potential role in treatment choice is not completely clarified. Indeed, still a relevant margin of uncertainty exists regarding the best adjuvant treatment for pT1cN0 or pT1N1mi HER2+ BCs.

In this context, the well-known biological heterogeneity of this disease might be a key factor significantly impacting long-term outcomes. HER2DX is a genomic assay that integrates clinical features (tumor size and nodal status) with gene expression signatures related to immune activation, luminal differentiation, tumor proliferation, and HER2 amplicon expression. It has shown the ability to more accurately predict response to neoadjuvant therapy and long-term outcomes in early-stage HER2-positive breast cancer^20–26^. In the APT trial, the HER2DX risk score identified a small subgroup of patients with a significantly higher risk of recurrence, potentially benefiting from more intensive therapy^12^. Similar findings were obtained in the ATEMPT trial and in a patient-level meta-analysis of 729 patients with stage 1 disease^13,27^. More recently, HER2DX risk-score has shown prognostic value in the RESPECT trial, a phase III trial that randomized patients of 70-80 yrs old with stage I-III disease to adjuvant trastuzumab +/− chemotherapy^28^. In this correlative analysis, HER2DX pCR-score showed a significant interaction with chemotherapy benefit in terms of overall survival, with high HER2DX pCR scores associated with greater benefit. Taken together, these data suggest that HER2DX low-risk patients might be safely treated with trastuzumab and paclitaxel (i.e., APT regimen), or potentially even with trastuzumab without chemotherapy for older patients, especially if presenting with small tumors (pT1a/pT1b) and if classified as pCR-low or pCR-medium by HER2DX. Future studies validating HER2DX in real-world cohorts are warranted, as they could provide valuable insights to further optimize risk stratification in early-stage HER2 + BC.

Beyond this, even if real-world studies confirm the effectiveness of the APT regimen in stage I HER2 + BC, one question remains open: is weekly paclitaxel chemotherapy and one year of trastuzumab truly necessary for all patients? In the APT trial, 68 patients (16.7%) had T1a tumors and 10 patients (2.5%) had T1mic tumors, with only two invasive disease-free events recorded in this group. In our cohort, 42 patients (15.3%) presented a pT1a tumor, and only one IBCFS event (a contralateral HER2- BC) was recorded in this patient subgroup. Indeed, the use of paclitaxel is not without toxicity. Treatment-induced peripheral neuropathy (TIPN) affects approximately a quarter of patients receiving this treatment, and in some cases, it can persist for months after the completion of therapy^29^. For these reasons, additional studies have been designed to investigate the possibility of maintaining efficacy outcomes while reducing potential toxicity profile in patients with early-stage HER2 + BC. For example, the phase II ATEMPT trial assessed the activity of adjuvant T-DM1 in patients with stage I HER2 + BC treated with upfront surgery. A 3-year IDFS of 97.8% (95% CI, 96.3–99.3) was observed with T-DM1, with a 46% incidence of clinically-relevant toxicities (a similar rate to those observed with the APT regimen, 47%); however, patients treated with T-DM1 presented less neuropathy^17^. The ATEMPT 2.0 trial is currently randomizing patients to the APT regimen or T-DM1 for 6 cycles (18 weeks) followed by trastuzumab for additional 11 cycles (NCT04893109). Moreover, the IRIS trial (NCT04383275) is currently assessing the potential efficacy of capecitabine as a chemotherapy partner for adjuvant trastuzumab in stage I HER2 + BC, and the single-arm phase II ADEPT trial is assessing the use of adjuvant endocrine therapy, pertuzumab, and trastuzumab in patients with HR + /HER2+ stage I BC (NCT04569747). In this context, the treatment of patients with low-risk HER2 + BC might potentially shift towards further de-escalated treatment regimens in future years.

Concluding, our study has some limitations. First, potential intrinsic biases in patient selection cannot be excluded, due to its retrospective design with a relatively limited follow-up period.

The present study cohort presents a median follow-up of 4.4 years. This potentially presents a significant limitation of the present study as it might limit the ability to capture late events or survival outcomes, leading to a possible underestimation of long-term outcomes. Therefore, future updates from this and other real-world studies reporting outcomes at a more prolonged follow-up are needed to further consolidate real-world evidence on the use of the APT regimen.

In addition, the small size of some patient subgroups should be considered while interpreting our results. It’s important to acknowledge that some of our results are based on very small subgroups within our cohort. Specifically, only 17 patients presented HER2 + BC stage IB, and 21 HER2 + BC stage IIA disease. Therefore, these observations should not be considered conclusive and require validation in other independent studies before final conclusions can be drawn.

Finally, data regarding chemotherapy-related toxicities could not be comprehensively assessed due to the retrospective nature of our analysis, and therefore, adverse events other than cardiac toxicity (e.g., neurotoxicity) have not been reported in this study, as we could not exclude relevant underreporting. The prospective assessment of the tolerability and safety profile of the APT regimen in real-world practice represents, therefore, an important objective for future studies.

Despite this, to our knowledge, this is the largest monocentric cohort to confirm the effectiveness of the APT regimen in real-world patients with early-stage HER2 + BC, selected using the same criteria of the APT. Moreover, our results suggest a need for caution in the use of this regimen for patients with micrometastatic nodal involvement (stage IB) or pT 2.1–3 cm tumors. Therefore, larger studies with longer follow-up are crucial to more thoroughly assess the safety and efficacy of this treatment approach in these rarer patient subgroups.

## Methods

### Study design and patient population

This monocentric retrospective study included patients diagnosed with early HER2 + BC and treated with the APT regimen at Istituto Oncologico Veneto (Padova) between January 2014 and October 2023.

In analogy to the APT trial, we included in this retrospective analysis adult females and males with a diagnosis of early HER2 + BC, characterized by tumor size T ≤ 3 cm and no lymph node involvement (the presence of nodal micrometastasis was permitted). HER2 positivity was defined as by clinical practice as a local immunohistochemistry score of HER2 3+ or a positive result in situ hybridization, according to ASCO/CAP guidelines^30^.

All patients included received at least one dose of adjuvant paclitaxel and trastuzumab according to APT regimen. Patients treated with regimens containing other cytotoxic or targeted agents (e.g., pertuzumab) were excluded. Patients with a diagnosis of contralateral BC, concomitant HER2-negative BC, prior neoadjuvant treatment for early BC, previous BC diagnosis or those previously treated with chemotherapy for other oncological conditions were excluded, as these conditions might significantly impact oncological outcomes and/or on-treatment toxicities.

In this patient population, cardiac function was, by clinical practice, routinely monitored by repeated cardiac ultrasound examinations in line with international guidelines^11^.

Demographic and clinicopathological characteristics, treatment and follow-up data were retrospectively collected from medical charts in a dedicated database.

Hormone receptor (HR) expression was determined by immunohistochemistry on primary tumor; positivity was defined as immunohistochemistry staining in at least 1% of tumor cells according to ASCO-CAP guidelines^31^.

Oncological outcomes were calculated according to STEEP definition^32^.

Recurrence-free survival (RFS) was defined as the time interval from first BC diagnosis to first distant recurrence, first local regional invasive recurrence, first invasive ipsilateral breast tumor recurrence or death from any cause, whichever occurred first.

Distant relapse-free survival (DRFS) was defined as the time interval from first BC diagnosis to first distant recurrence or death from any cause, whichever occurred first.

Invasive breast cancer–free survival (IBCFS) was defined as the time interval from first BC diagnosis to first distant recurrence, first local regional invasive recurrence, first invasive ipsilateral or controlateral breast tumor recurrence or death from any cause, whichever occurred first.

Patients alive without event at the time of analysis were censored at the date of last follow-up.

### Statistical analysis

#### Statistical analysis was performed using IBM SPSS Statistics 29.0

The clinicopathological characteristics were summarized using standard descriptive statistics. Kaplan–Meier method was used to estimate RFS, DRFS, and IBCFS and and reported with its 95% confidence intervals (95% CIs). Log-rank test was used to compare outcomes between groups.

For the evaluation of prognostic factors, univariate Cox regression modeling for proportional hazards was used to calculate hazard ratios and their 95% CI. All reported *p*-values are two-sided, and the significance level was set at 5% (*p* < 0.05).

The study was reviewed and approved by the Ethics Committee of Istituto Oncologico Veneto (Internal Code: EM 2025-11) and conducted in accordance with the Declaration of Helsinki. Written informed consent was obtained from participants when needed according to the involved site legislation.

## Supplementary information

Supplementary Information

## Data Availability

The datasets that support the findings of this study are not publicly available in compliance with EU privacy regulations. Data can be made available through a request to the corresponding author after fulfillment of legal/ethical requirements. Further information is available from the corresponding author upon request.
