# Supplementary material for: Real-world outcomes of early-stage HER2-positive breast cancer patients treated with adjuvant paclitaxel and trastuzumab
Source: NPJ Breast Cancer. 2025 Nov 26;11:132. doi: 10.1038/s41523-025-00846-4 (PMC12657504; doi:10.1038/s41523-025-00846-4)
Supplement: Supplementary file 1 — Supplementary Information [file 41523_2025_846_MOESM1_ESM.pdf]

Supplementary data

Supplementary Figure 1. Kaplan-Meier curve showing invasive breast cancer-free survival (dashed line highlighting 36 and 60 months).

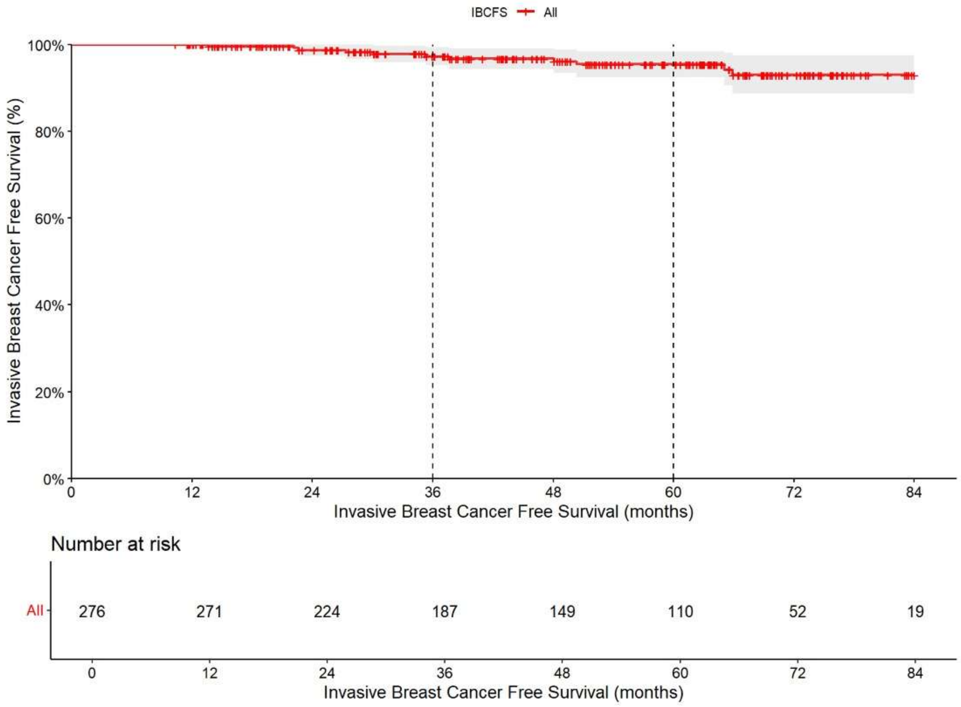

Supplementary Figure 2. Kaplan-Meier curves showing recurrence-free survival and distant relapse-free survival (dashed line highlighting 36 and 60 months) according to menopausal state (a-b), to grade (c-d) and to HER2 status assessed by immunohistochemistry (e-f). a Kaplan-Meier curve reporting RFS according to menopausal state. Pre-menopausal state in blue, post-menopausal state in red**. b** Kaplan-Meier curve reporting DRFS according to menopausal state. Pre-menopausal state in blue, post-menopausal state in red. **c.** Kaplan-Meier curve reporting RFS according to grade. Grade 1-2 in blue, Grade 3 in red**. d.** Kaplan-Meier curve reporting DRFS according to grade. Grade1-2 in blue, Grade 3 in red. **e.** Kaplan-Meier curve reporting RFS according to HER2 status. HER2 2+/amplifield in blue, HER2 3+ in red. **f.** Kaplan-Meier curve reporting DRFS according to HER2 status. HER2 2+/amplifield in blue, HER2 3+ in red.

a.

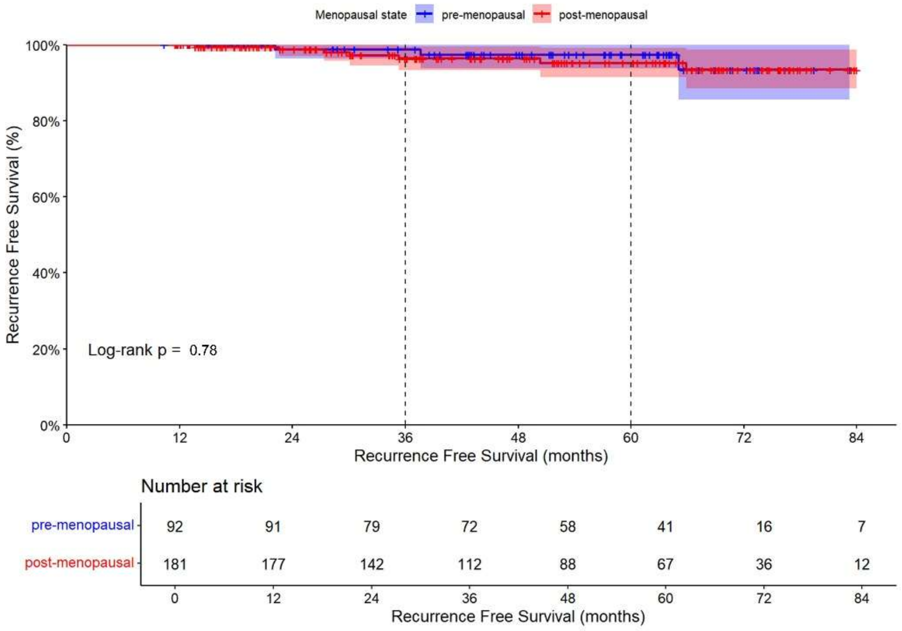

b.

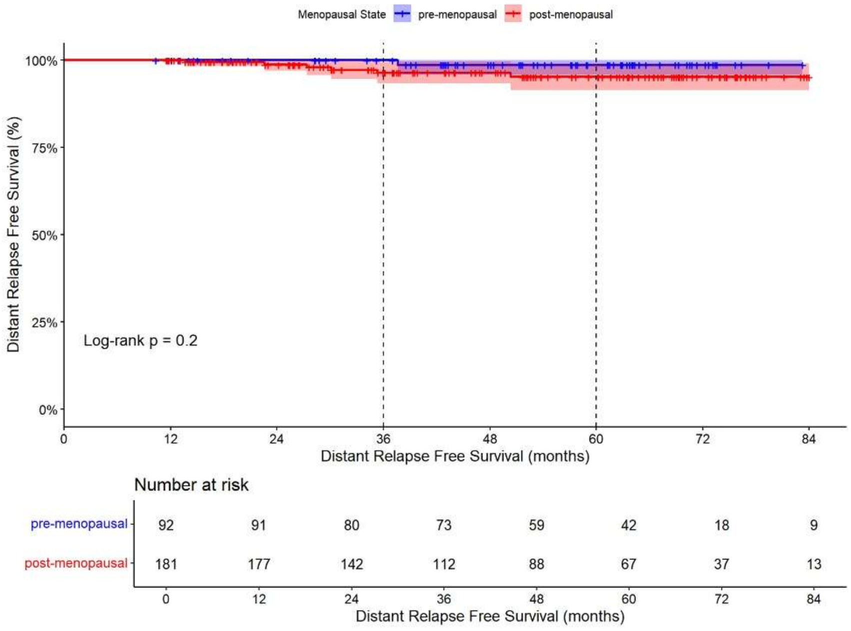

c.

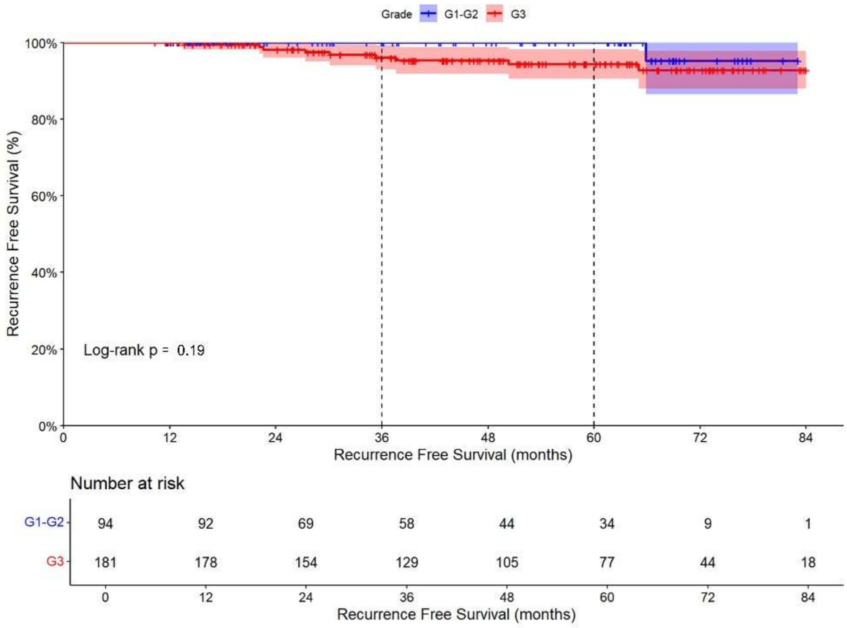

Grade G1-G2 G3
100%
80%
60%
40%
20%
0%
Recurrence Free Survival (%)
Log-rank p = 0.19
0 12 24 36 48 60 72 84
Recurrence Free Survival (months)
Number at risk
G1-G2 94 92 69 58 44 34 9 1
G3 181 178 154 129 105 77 44 18
0 12 24 36 48 60 72 84
Recurrence Free Survival (months)

d.

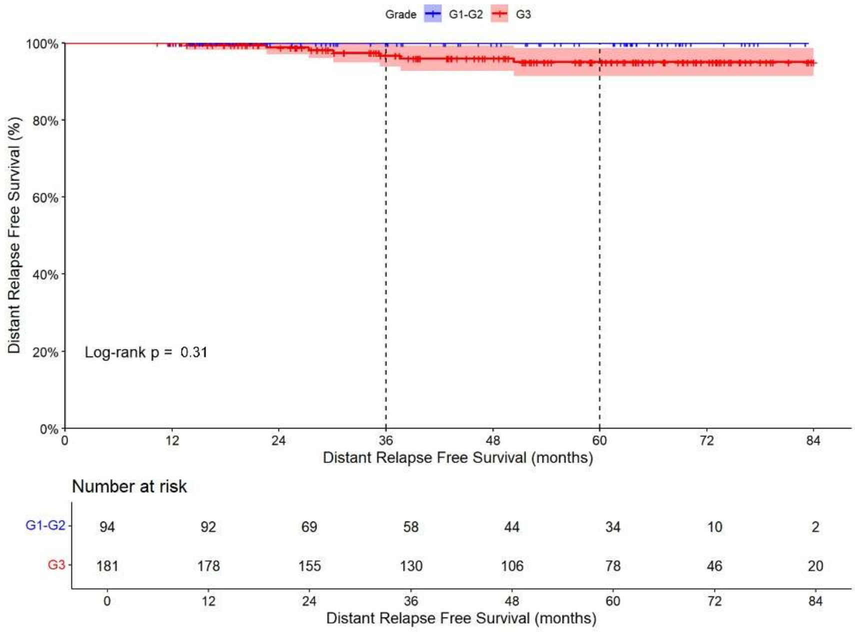

Grade G1-G2 G3
100%
80%
60%
40%
20%
0%
Distant Relapse Free Survival (%)
Log-rank p = 0.31
0 12 24 36 48 60 72 84
Distant Relapse Free Survival (months)
Number at risk
G1-G2 94 92 69 58 44 34 10 2
G3 181 178 155 130 106 78 46 20
0 12 24 36 48 60 72 84
Distant Relapse Free Survival (months)

e.

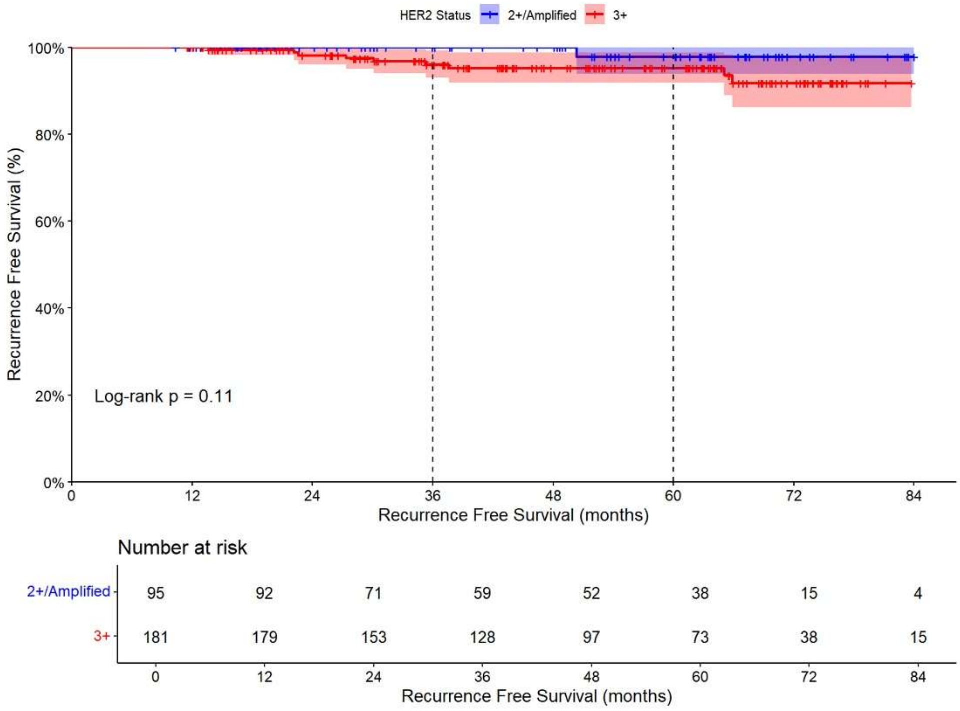
HER2 Status 2+/Amplified 3+
100%
80%
60%
40%
20%
0%
Recurrence Free Survival (%)
Log-rank p = 0.11
0 12 24 36 48 60 72 84
Recurrence Free Survival (months)
Number at risk
2+/Amplified 95 92 71 59 52 38 15 4
3+ 181 179 153 128 97 73 38 15
0 12 24 36 48 60 72 84
Recurrence Free Survival (months)

f.

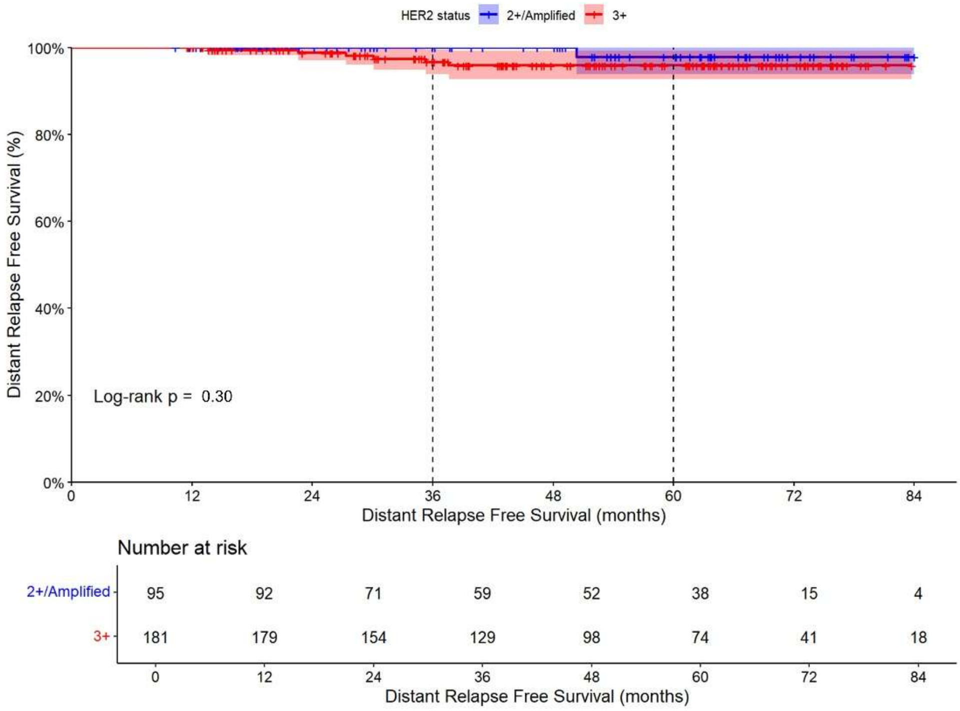
HER2 status 2+/Amplified 3+
100%
80%
60%
40%
20%
0%
Distant Relapse Free Survival (%)
Log-rank p = 0.30
0 12 24 36 48 60 72 84
Distant Relapse Free Survival (months)
Number at risk
2+/Amplified 95 92 71 59 52 38 15 4
3+ 181 179 154 129 98 74 41 18
0 12 24 36 48 60 72 84
Distant Relapse Free Survival (months)

Supplementary Figure 3. Kaplan-Meier curves showing recurrence-free survival and distant relapse-free survival (dashed line highlighting 36 and 60 months) in accordance with the tumor size (pT) measured in centimeters. a Kaplan-Meier curve reporting RFS according to tumor size (pT), pT ≤ 1 cm in blue, pT > 1 cm in red**. b.** Kaplan-Meier curve reporting DRFS according to tumor size (pT), pT ≤ 1 cm in blue, pT >1 cm in red.

a.

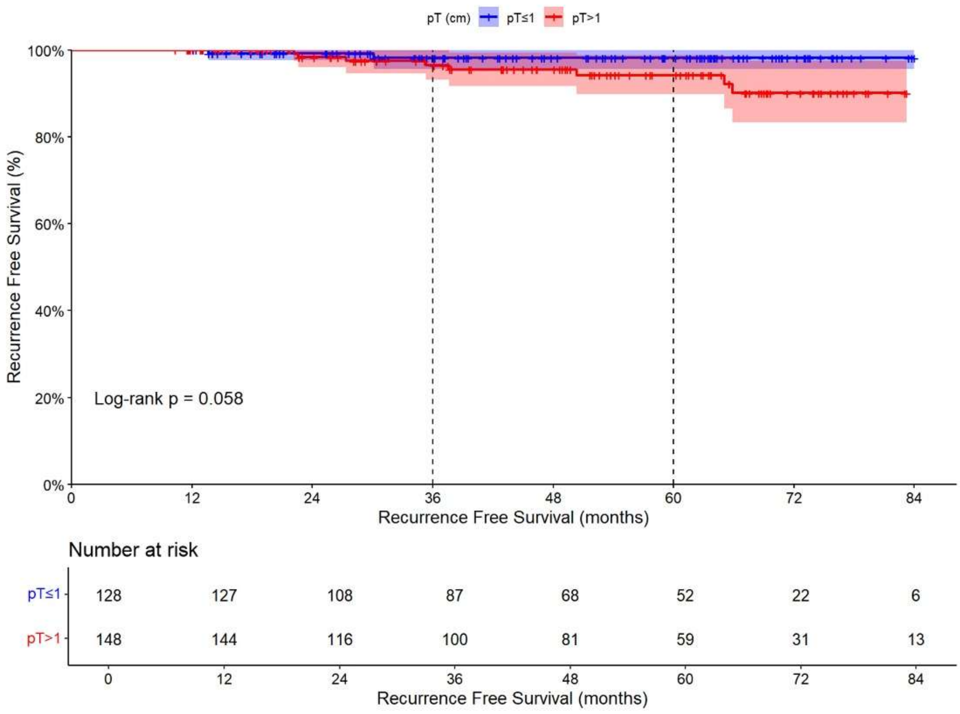

b.

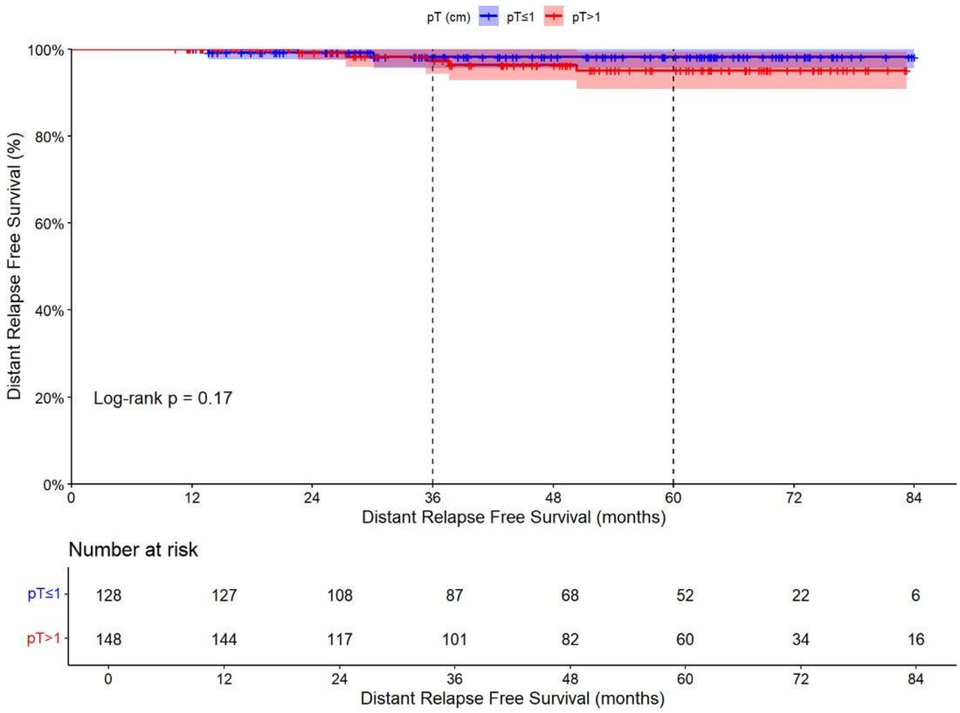

Supplementary Figure 4. Kaplan-Meier curves showing recurrence-free survival and distant relapse-free survival (dashed line highlighting 36 and 60 months) in accordance with the tumor size (pT) in the subgroup of patients with tumours up to 2 cm. a. Kaplan-Meier curve reporting RFS according to tumor size (pT), pT ≤ 1 cm in blue, pT > 1 cm ≤ 2 cm in red. **b.** Kaplan-Meier curve reporting DRFS according to tumor size (pT), pT ≤ 1 cm in blue, pT > 1 cm ≤ 2 cm in red.

a.

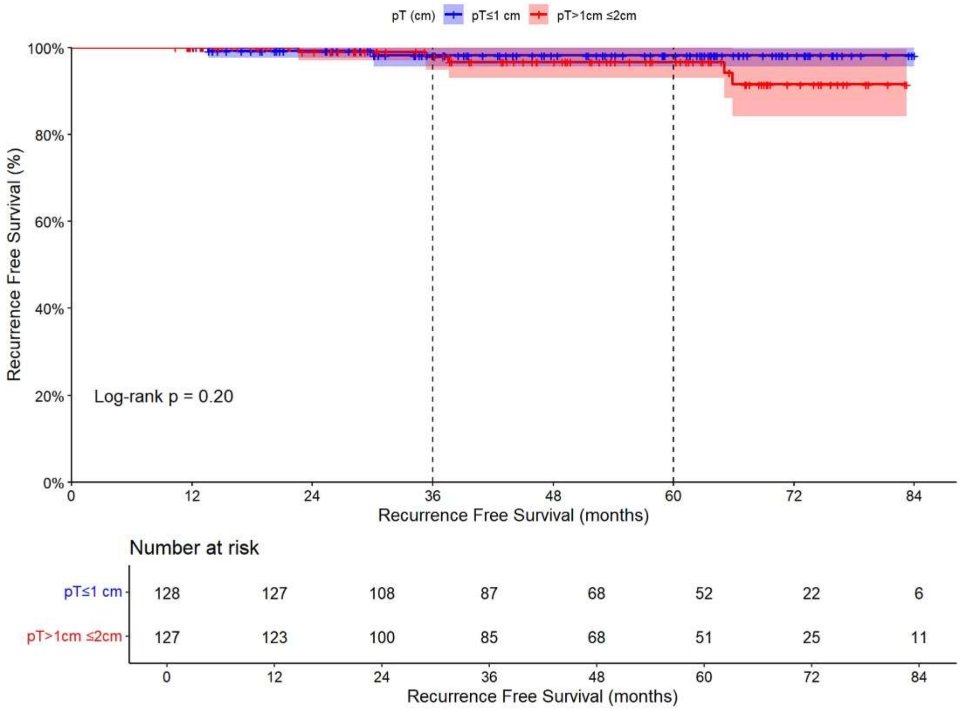

b.

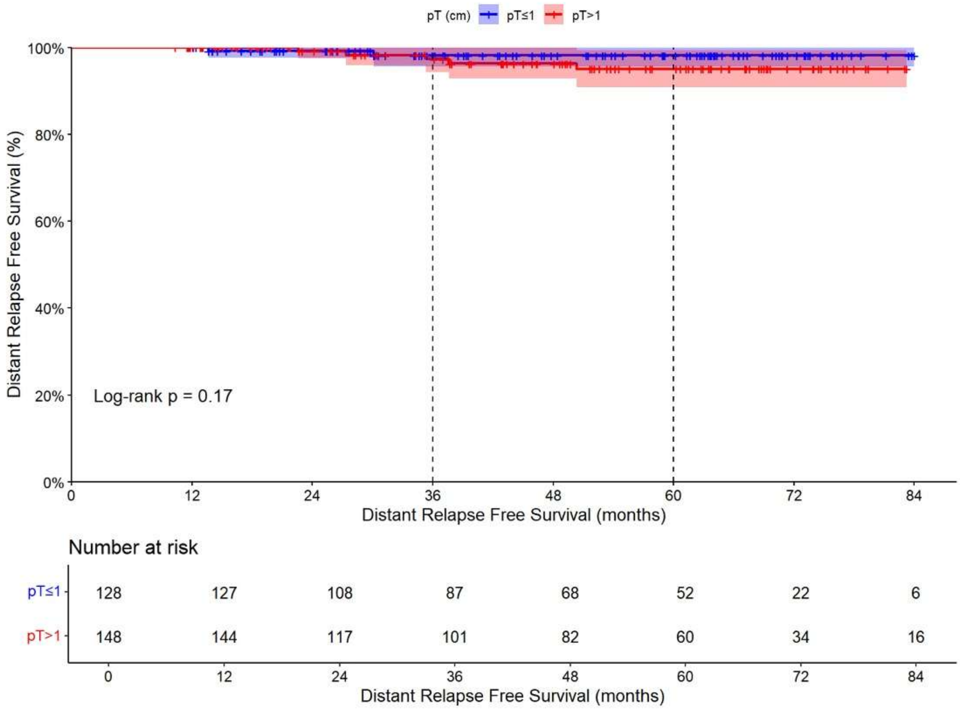

Supplementary Table 1. Cardiac toxicity

Patient	Event Description	LVEF basal (%)	Timepoint at which cardiac toxicity was identified (months)	LVEF decreased (%)	Clinical evolution	LVEF at 3 weeks (%)	LVEF at 6 weeks (%)	Final outcome
1	Heart failure grade 3 (pulmonary oedema)	68%	3	71%	Pulmonary oedema resolved with pharmacological therapy	58%	NA^	AE resolved without sequelae
2	Symptomatic decrease in LVEF (absolute decline of ≥16% from baseline associated with dyspnea)	74%	9	47%	Dyspnea resolved with pharmacological therapy	49%	48%	AE resolved without sequelae (LVEF increased to 60% at subsequent evaluation)
3	Asymptomatic decrease in LVEF (absolute decline of ≥10% from baseline to below 55%)	61%	9	51%	No clinical symptoms	59%	NA^	AE resolved without sequelae (Trastuzumab not restarted due to patient/clinician decision)
4	Asymptomatic decrease in LVEF (absolute decline of ≥10% from baseline to below 55%)	60%	3	50%	No clinical symptoms	55%	NA^	AE resolved without sequelae (Trastuzumab not restarted due to patient/clinician decision)
5	Asymptomatic decrease in LVEF (absolute decline of ≥10% from baseline to below 55%)	56%	9	46%	No clinical symptoms	42%	52%	AE resolved without sequelae

TH: weekly paclitaxel and trastuzumab
H:
Herceptin
^ LVEF evaluation at week 6 only performed if LVEF decrease not resolved at week 3
